# Assessment of the risk of discontinuation of tenofovir disoproxil fumarate after delivery and the benefit of continued treatment in patients with immune tolerance

**DOI:** 10.3389/fmed.2025.1597664

**Published:** 2025-11-07

**Authors:** Huan Liang, Hengkai Liang, Bobin Hu, Long Huang, Hongqian Liang, Minghua Su, Rongming Wang, Tumei Su, Qingmei Li, Yanfei Feng, Jianning Jiang

**Affiliations:** 1 Department of Infectious Diseases, The First Affiliated Hospital of Guangxi Medical University, Nanning, China; 2 Key Laboratory of Early Prevention and Treatment for Regional High Frequency Tumor (Guangxi Medical University), Ministry of Education, Nanning, China; 3 Guangxi Key Laboratory of Early Prevention and Treatment for Regional High Frequency Tumor, Nanning, China

**Keywords:** immune tolerance phase, tenofovir disoproxil fumarate, discontinuation, risk, benefit

## Abstract

**Background:**

Both domestic and foreign guidelines recommend that chronic hepatitis B virus (HBV) infection pregnant women in the immune tolerance phase (ITP) discontinue antiviral therapy after delivery, this study aimed to investigate the risk of postpartum drug withdrawal and the pros and cons of continuing treatment of tenofovir dipivoxil fumarate (TDF) in ITP pregnant women.

**Methods:**

The study group consisted of 116 naive pregnant women in ITP, the control group included 81 naive chronic hepatitis B (CHB) pregnant women with HBeAg-positive and high viral load. The study aimed to compare the risk of discontinue rebound within 48 weeks postpartum, the antiviral efficacy and bone and renal safety of continuing TDF treatment until 144 weeks postpartum between the two groups.

**Results:**

There was no significant difference in the reduction of HBV DNA levels between the ITP group and the CHB group prior to labor (*p* < 0.05), with a 100% success rate in mother-to-child transmission prevention for both cohorts. Postpartum, 38.8% (45/116) and 18.5% (15/81) of parturients in the ITP group and CHB group, respectively, discontinued TDF at various time intervals. Comparative analysis of the risk of viral rebound within 48 weeks postpartum revealed no significant difference between the two groups (*p* > 0.05). The ITP group had higher rates of suboptimal response and low viremia occurrence compared to the CHB group (*p* < 0.05) at 48 weeks postpartum, but following salvage therapy up to 144 weeks postpartum, the cumulative rate of complete virological response(CVR) in the ITP group was non-inferior to that in the CHB group (*p* > 0.05). There were no significant differences in the average eGFR and serum phosphorus levels between the two groups from baseline to 144 weeks after TDF treatment.

**Conclusion:**

Postpartum discontinuation of TDF poses significant risks for immunotolerant pregnant women, whereas continuing TDF treatment for 144 weeks postpartum demonstrates favorable antiviral efficacy, bone and renal safety profiles.

## Introduction

Hepatitis B virus infection is a significant global public health issue, with mother-to-child transmission (MTCT) being a major route of transmission in China (1), antiviral prophylaxis during pregnancy in HBV-infected women is beneficial for disease control, prevention of vertical transmission, and ensuring maternal and neonatal safety (2), these efforts play a crucial role in achieving the World Health Organization’s global health sector strategy goal of eliminating viral hepatitis by 2030 (3). Currently, there is consensus on using pregnancy category B nucleotide analogues (NAs) for antiviral therapy in immunotolerant pregnant women with high viral loads. However, it is commonly believed that discontinuing therapy postpartum in immunotolerant mothers does not result in significant harm, and continuing therapy postpartum offers limited efficacy and increases the risk of resistance (4, 5). Therefore, guidelines both domestically and internationally suggest that immunotolerant pregnant women can discontinue therapy postpartum. However, in the current trend of expanding antiviral therapy for chronic HBV infection, there is a redefinition of indications for antiviral treatment in immunotolerant patients with normal ALT levels and high viral loads, especially those over 30 years old or with a family history of cirrhosis or hepatocellular carcinoma. This contradicts recommendations for postpartum therapy discontinuation in guidelines. Our research team believes it is necessary to assess the risks of postpartum discontinuation and the efficacy and safety of continuing treatment postpartum in immunotolerant pregnant women, to provide scientific evidence for clinical treatment decisions.

## Materials and methods

### Ethics approval and consent to participate

This study was approved by the Ethics Committee of the First Affiliated Hospital of Guangxi Medical University, with ethics approval number No. 2022-KY-(061). To ensure adherence to ethical standards, all research protocols were conducted in accordance with the guidelines outlined in the 1975 Helsinki Declaration, which was revised in 2013. Written informed consent was obtained from all participating patients.

### Study patients

This study is a retrospective study enrolled a cohort of pregnant women with chronic HBV infection receiving TDF therapy, recruited from the outpatient clinic of the Department of Infectious Diseases, First Affiliated Hospital of Guangxi Medical University from January 2012 to December 2022. Inclusion criteria were as follows: (1) ITP group: HBsAg-positive, HBeAg-positive, HBV DNA > 2 × 10^7^ IU/ml, and ALT within normal limits; (2) CHB group: HBsAg-positive, HBeAg-positive, HBV DNA > 2 × 10^5^ IU/ml, with persistent or intermittent ALT elevation (> 50 U/L); (3) Initiation of TDF for antiviral prophylaxis or treatment during pregnancy, with follow-up extended to 48 weeks postpartum; (4) Continued follow-up to 144 weeks postpartum if complete virological response was not achieved. Exclusion criteria included: (1) Pregnant women previously treated with TDF or other NAs (e.g., LdT, TAF, ETV); (2) Pregnancy complications or organic damage other than HBV infection; (3) Pregnant women with concurrent viral infections other than HBV; (4) Pregnant women with chronic liver diseases such as alcoholic liver disease or cirrhosis. Patients in the ITP discontinuation group voluntarily ceased TDF therapy for more than 4 weeks postpartum within 0–12 weeks after delivery, in accordance with guidelines for CHB. Patients in the CHB discontinuation group were those who discontinued TDF therapy for more than 4 weeks within the same postpartum period without medical advice.

### Study design

**FIGURE 1:**
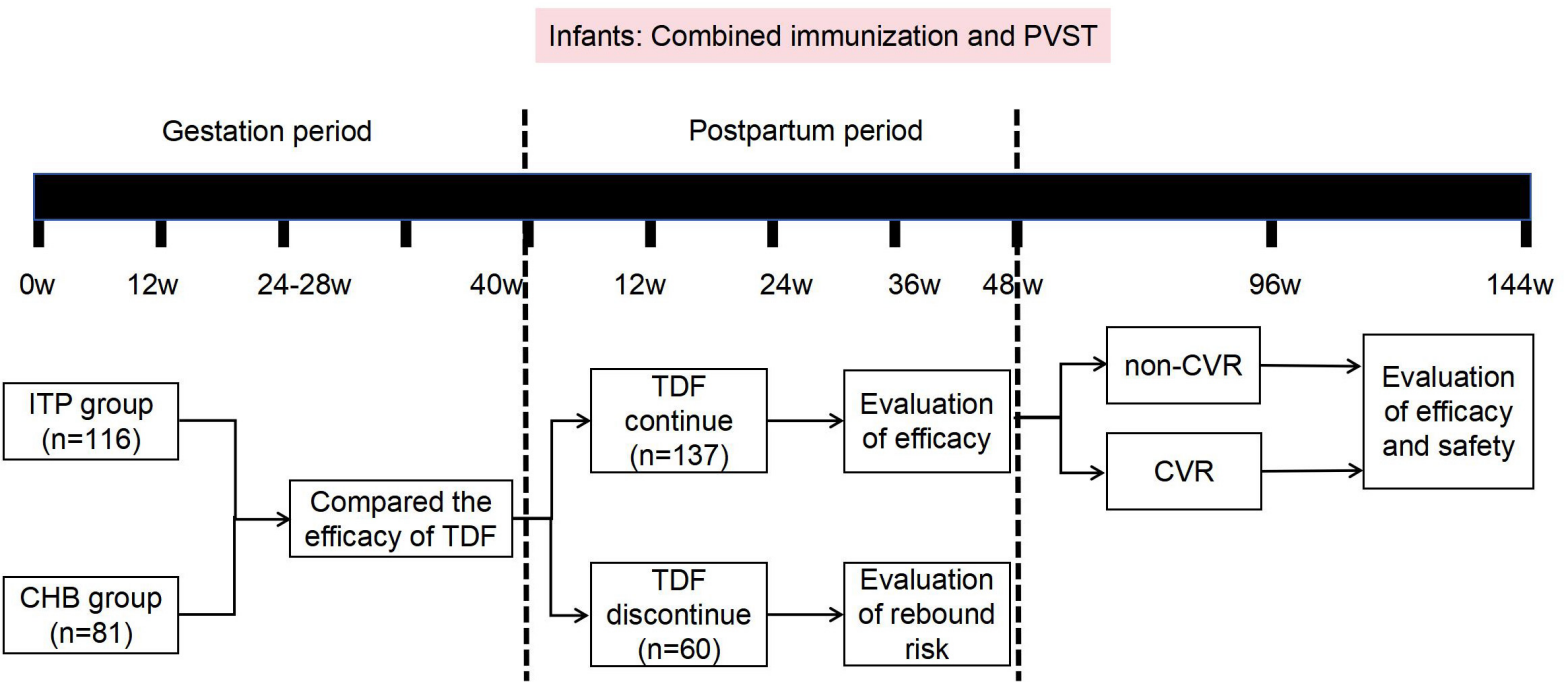
Research flowchart. ITP, immune tolerence phase; CHB, chronic hepatitis B; TDF, tenofovir dipivoxil fumarate; PVST, postpartum virological serological testing.

Patients in both groups initiated antiviral therapy during pregnancy. They took 300 mg of TDF daily, medication compliance was monitored through regular tablet counting, with monitoring conducted every 3 months for HBV DNA, HBV serological markers, liver and kidney function, blood phosphate, and AFP levels, and every 6 months with liver ultrasound examinations until 48 weeks postpartum. Follow-up was continued until 144 weeks postpartum for those who did not achieve complete virological response. Newborns received immunoglobulin injection at birth along with full hepatitis B vaccination, and were followed up for HBsAg testing at 7–12 months of age to assess MTCT prevention. The study roadmap is illustrated in Figure 1.

### Statistical analysis

Data analysis was performed using SPSS26.0 statistical software (IBM, Armonk, NY, United States), and the pictures were plotted by GraphPad Prism8.0 software. For normally distributed metric data, mean ± standard deviation ($\bar{\chi }$ ± s) was used, while skewed metric data was expressed as median (M) (P25∼P75) or M (IQR). For inter-group comparisons of continuous variables that met normality and variance homogeneity assumptions, independent sample *t-*tests were employed. In case of heteroscedasticity, a *t-*test was used. One-way analysis of variance (ANOVA) and Kruskal-Wallis tests were used for inter-group comparisons, while R*C cross-table chi-squre test (χ^2^)was used for inter-group comparisons of unordered categorical variables. Statistical significance was set at *p* < 0.05. Comparisons of cumulative CVR rates between groups were performed using the Kaplan-Meier method and assessed with the Log-rank test. Additionally, the hazard ratio (HR) and its 95% confidence interval (CI) were calculated to quantify the risk difference in achieving CVR between the CHB and ITP groups.

## Results

### General characteristics of the study subjects

This study enrolled a total of 197 pregnant women with chronic HBV infection initiating antiviral therapy with TDF during pregnancy. The study group comprised 116 immunotolerant pregnant women, among whom 79.3% (92/116) were aged over 30 years or had a family history of hepatitis B-related liver cirrhosis or hepatocellular carcinoma. The control group included 81 pregnant women with HBeAg-positive and high viral load chronic hepatitis B. General patient characteristics are summarized in Table 1.

**TABLE 1:** The general information of chronic hepatitis B virus (HBV) infection in pregnant women.

Observational content	ITP group (n = 116)	CHB group (n = 81)
Gestational age (years), ($\bar{\chi }\pm$ s)	30.03 ± 4.25	30.09 ± 3.40
Family history of hepatitis B (%)	54.3% (63/116)	60.5% (49/81)
Family history of cirrhosis and liver cancer (%)	11.2% (13/116)	17.3% (14/81)
Antiviral gestational age (weeks), ($\bar{\chi }\pm$ s)	25.51 ± 2.89	22.33 ± 6.47
Baseline HBVDNA (log_10_ IU/ml), ($\bar{\chi }\pm$s)	8.08 ± 0.39	7.48 ± 1.03
Baseline ALT (U/L), M (P25∼P75)	17 (12∼23)	50 (25∼121)
Course of TDF treatment until parturient (weeks), M (P25∼P75)	14.71 (12.00∼15.79)	15.07 (11.67∼19.03)
Postpartum withdrawal cases (*n*, %)	45 (38.8%)	15 (18.5%)
Treated until 48 weeks postpartum cases (*n*, %)	71 (61.2%)	66 (81.5%)

### Risk study of TDF discontinuation postpartum in immunotolerant patients

**FIGURE 2:**
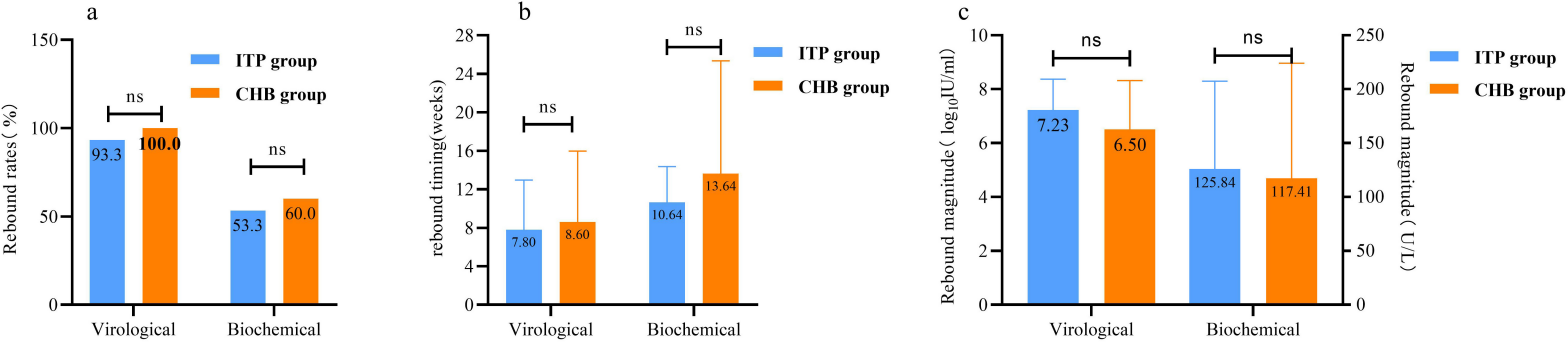
Comparison of the risk of rebound after tenofovir dipivoxil fumarate (TDF) withdrawal in 48 weeks postpartum between immune tolerence phase (ITP) group and chronic hepatitis B (CHB) group. **(a)** The comparison of withdrawal rebound rates. **(b)** The comparison of drug withdrawal rebound in time. **(c)** The comparison of drug withdrawal rebound level. Virological rebound: patients who discontinue TDF treatment, with hepatitis B virus (HBV) DNA levels rising by > 1 log_10_ IU/ml from the lowest point during treatment or at cessation, or converting from negative to positive and confirmed by repeat testing with the same assay 1 month later, with or without alanine aminotransferase (ALT) elevation. Biochemical rebound: patients on TDF therapy or after cessation, where ALT levels, having previously been below the limit of detection (9–50 U/L), rise again without other causes for ALT elevation, with or without HBV DNA increase. ns *p* > 0.05.

Following delivery, 38.8% (45/116) of immunotolerant patients and 18.5% (15/81) of chronic hepatitis B patients discontinued TDF at various time points. Subsequent follow-up continued until 48 weeks postpartum to compare virological and biochemical rebound rates (Figure 2a), rebound timing (Figure 2b), and rebound magnitude (Figure 2c) between the two groups, there was no significant difference in the risk of TDF discontinuation between the two groups of mothers.

Further subgroup analysis of postpartum withdrawal among women in the ITP group (*n* = 45) revealed no significant differences in rebound risk between immediate withdrawal and withdrawal at 4–12 weeks postpartum, or between women aged ≤ 30 and > 30 years (*p* > 0.05),the results are shown in Table 2.

**TABLE 2:** The risk of postpartum discontinuation of tenofovir dipivoxil fumarate (TDF) in immunentolerant women.

Observational content	Grouping by postpartum cessation time			Grouping by age at cessation		
	0-week group (n = 40)	4–12-week group (n = 5)	*P*-value	≤30 years group (n = 24)	>30 years group (n = 21)	*P*-value
Virological rebound rate (*n*, %)	87.5 (35/40)	100.0 (5/5)	0.539	91.7 (22/24)	95.2 (20/21)	0.551
Virological rebound time (weeks), M (IQR)	6.0 (8.0)	6.0 (10.0)	0.881	8.0 (8.0)	4.0 (5.0)	0.182
Virological rebound level (log_10_ IU/ml), M (IQR)	8.0 (1.0)	8.0 (2.5)	0.916	8.0 (1.0)	8.0 (1.0)	0.685
Biochemical rebound rate (*n*, %)	55.0 (22/40)	40.0 (2/5)	0.435	58.3 (14/24)	52.4 (11/21)	0.460
Biochemical rebound time (weeks), M (IQR)	3.5 (9.5)	12.0 (4.0)	0.392	4.0 (9.0)	4.0 (10.0)	0.481
Biochemical rebound ALT level (U/L), M (IQR)	86.5 (86.5)	60.5 (7.0)	0.195	87.0 (84.0)	67.0 (96.0)	0.289

### Study on the benefits of continued TDF therapy in immune tolerant period patients postpartum

**FIGURE 3:**
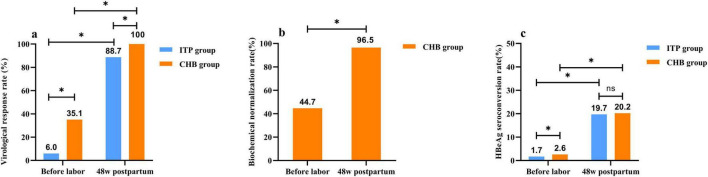
Two groups of patients with tenofovir dipivoxil fumarate (TDF) treatment to the curative effect of postpartum 48 weeks compared with before delivery. **(a)** The comparism of virological response rate, virological response was defined as achieving hepatitis B virus (HBV) DNA < 100 IU/ml after receiving TDF with good adherence, continuing treatment for 48 weeks or longer postpartum. **(b)** The comparism of biochemical normalization rate, biochemical normalization referred to the decrease in elevated alanine aminotransferase (ALT) levels to the detection limit after treatment initiation or during the course of TDF therapy. **(c)** The comparism of HBeAg seroconversion rate, HBeAg seroconversion denoted the disappearance of HBeAg in previously HBeAg-positive patients, accompanied by the appearance of HBeAb.*p < 0.05.ns *p* > 0.05.

In pregnant women treated with TDF until peripartum, the average duration of antiviral therapy was shorter in the ITP group compared to the CHB group (*p* < 0.05). Prior to delivery, 81.0% (94/116) of pregnant women in the ITP group and 91.4% (74/81) in the CHB group achieved viral load reduction to low levels (HBV DNA ≤ 2 × 10^5^ IU/ml). There was no significant difference in the extent of viral load reduction between the two groups prior to delivery [(3.81 ± 0.89) vs. (3.88 ± 1.29) log_10_ IU/ml, *p* > 0.05]. Both groups achieved a 100% rate of preventing MTCT. At 48 weeks postpartum with TDF treatment, both groups showed higher virological response rates, HBeAg seroconversion rates, and normalization of ALT compared to pre-delivery levels. The postpartum virological response rate in the ITP group was lower than that in the CHB group, with no statistically significant difference observed in HBeAg seroconversion rates between the two groups at 48 weeks postpartum. Detailed results are presented in Figure 3.

### Safety of postpartum TDF treatment in immunotolerant patients

**FIGURE 4:**
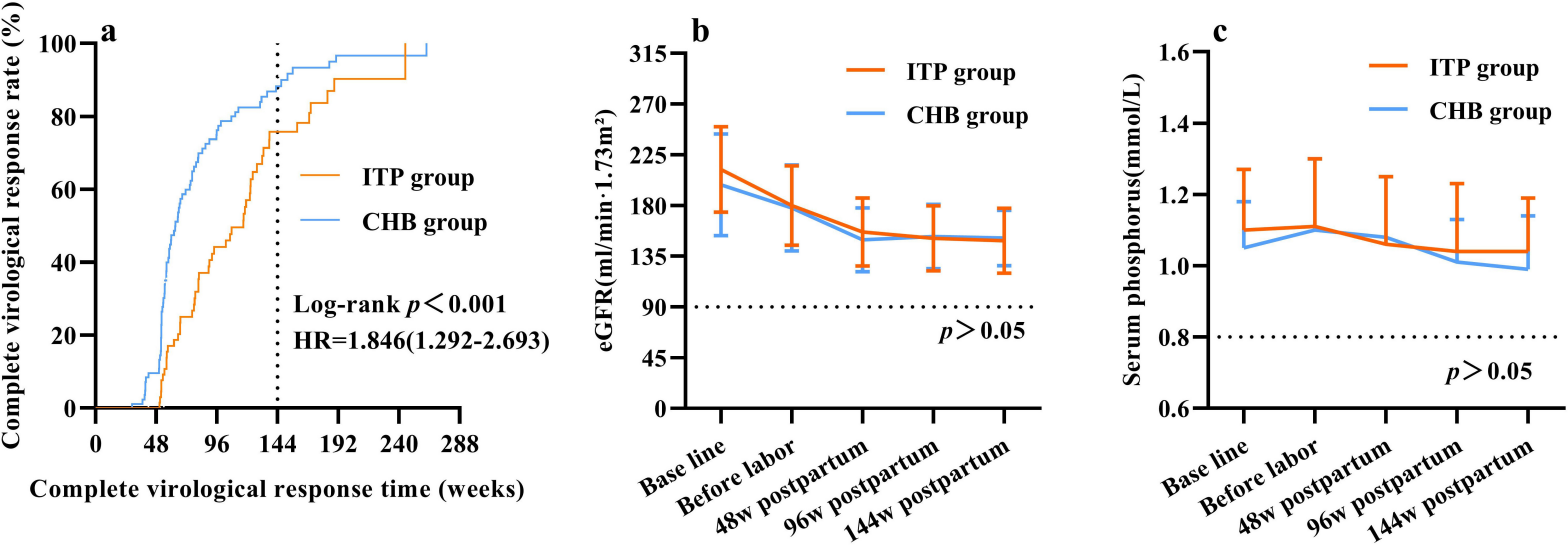
Safety study of postpartum continuation of tenofovir dipivoxil fumarate (TDF) up to 144 weeks in two groups. **(a)** Cumulative complete virologic response rate. “Time” was defined as the number of weeks from the initiation of TDF antiviral therapy until the achievement of a complete virological response. Data for subjects who did not achieve complete virological response (CVR) by the end of the study period were censored at their last follow-up visit. **(b)** Changes in eGFR levels. **(c)** Changes in serum phosphorus level.

At 48 weeks postpartum, no cases of virological breakthrough were observed in either group under TDF treatment. The ITP group exhibited lower virological response rates compared to the CHB group, with higher rates of suboptimal response and low viremia occurrence (Table 3). Patients with suboptimal response and low-level viremia in both groups were subjected to salvage therapy with TDF combined with entecavir (ETV) or switched to Tenofovir Amibufenamide (TMF). Survival analysis for CVR demonstrated that the CHB group had a significantly higher probability of achieving CVR compared to the ITP group [Hazard Ratio (HR) = 1.846, 95% CI: 1.292–2.693; Log-rank *p* < 0.001]. By 144 weeks postpartum, the time to achieve complete virological response (CVR) was later in the ITP group compared to the CHB group (Figure 4a). The cumulative rate of complete virological response (HBV DNA < 20 IU/mL) was lower in the ITP group [92.0% (46/50) vs. 98.3% (59/60), *p* > 0.05] than in the CHB group. However, with extended treatment duration, the cumulative complete virological response rate gradually increased and approached equivalence.

**TABLE 3:** Comparison of virological response of tenofovir dipivoxil fumarate (TDF) treatment to 48 weeks postpartum.

Observational content	ITP group (n = 71)	CHB group (n = 66)	*P*-value
Virological breakthrough rate (%)	0.0% (0/71)	0.0% (0/66)	–
Suboptimal response rate (%)	18.3% (13/71)	1.5% (1/66)	0.000
Virological response rate (%)	81.7% (58/71)	98.5% (65/66)	0.010
Low viremia rate (%)	32.7% (16/49)	4.5% (3/66)	0.000

Virological response: tenofovir dipivoxil fumarate (TDF) treatment showed good compliance, achieving hepatitis B virus (HBV) DNA < 100 IU/ml by 48 weeks postpartum. Virological breakthrough: under good compliance with TDF treatment, virological breakthrough was defined as an increase in HBV DNA levels > 1 log_10_ IU/ml from the lowest recorded level during treatment without treatment modification, or reversion from negative to positive HBV DNA, confirmed by repeat testing with the same assay 1 month later, with or without alanine aminotransferase (ALT) elevation. Suboptimal response: good compliance with TDF treatment resulted in HBV DNA > 100 IU/mL at 48 weeks postpartum. Low viremia: good compliance with TDF treatment resulted in HBV DNA levels between 20 and 100 IU/mL at 48 weeks postpartum and beyond.

There was no significant difference observed in the decline trend of eGFR levels between the groups, with both showing a decline in eGFR levels compared to baseline at 48 weeks postpartum (*p* < 0.05). Further follow-up at 96 and 144 weeks postpartum indicated no significant decline in eGFR levels compared to those at 48 weeks postpartum (Figure 4b). Comparison of serum phosphate levels between the ITP and CHB groups showed no significant differences in decline trends. At 48, 96, and 144 weeks postpartum, serum phosphate levels did not differ significantly from baseline levels (Figure 4c).

## Discussion

High viral load of HBV is an independent risk factor for disease progression to liver cirrhosis, liver failure, and hepatocellular carcinoma in chronic HBV-infected patients (6), and remains the predominant risk factor for HBV MTCT (7). The population of immunotolerant patients is substantial (8), with those harboring high HBV viral loads being a priority group for MTCT prevention strategies (9). This study demonstrates that antiviral therapy with TDF during pregnancy significantly reduces maternal HBV DNA levels in both immunotolerant and chronic HBV-infected pregnant women with high viral loads, effectively achieving the goal of preventing MTCT. Clinically, a significant proportion of immunotolerant patients experience an indeterminate phase (10), with liver histopathology indicating progression (11, 12). Multiple studies indicate that chronic HBV-infected individuals with normal alanine aminotransferase (ALT) levels remain at risk of disease progression (13, 14). Immunotolerant patients who do not receive antiviral therapy face significantly higher risks of hepatocellular carcinoma (HCC), mortality, or transplantation compared to those regularly treated with NAs following HBV activity suppression (15, 16), underscoring the necessity of antiviral therapy in this patient population. This study confirms that postpartum continuation of TDF treatment among immunotolerant mothers achieves favorable antiviral efficacy, consistent with findings by Feng et al. (17).

However, guidelines both domestically and internationally generally assert that immunotolerant patients of HBV show no hepatic activity and thus do not meet indications for antiviral therapy. Furthermore, antiviral efficacy in these patients is inferior to that in those with chronic hepatitis B, with long-term medication increasing the risk of resistance. Advocating for discontinuation of therapy postpartum in immune-tolerant phase mothers has been proposed (7, 18-20). A prospective study of 330 pregnant women in a Chinese cohort showed that stopping tenofovir immediately at delivery did not increase the risk of virological relapse and retreatment compared with a longer duration of tenofovir treatment, suggesting that shortening the duration of peripartum antiviral prophylaxis from 12 weeks to immediately after delivery can be considered (4). However, all the subjects in this study were HBsag-positive pregnant women, and no comparison between immune tolerance and chronic hepatitis B groups was performed. Our study reveals that the risk of rebound after discontinuation is comparable between immunotolerant patients and those with CHB. For immunotolerant patients, regardless of immediate postpartum cessation or cessation at 4–12 weeks, and whether age at cessation is ≤ 30 years, the risk of rebound did not statistically differ, consistent with findings by Zhang et al. (21). This suggests that discontinuation of therapy postpartum in immune-tolerant phase mothers, even those who received antiviral prophylaxis during pregnancy to prevent vertical transmission, is unsafe.

Moreover, to achieve the goal of eliminating viral hepatitis as a public health threat by 2030, the treatment paradigm for chronic hepatitis B is shifting from “Treat only if…” to “Treat all…” (22-25). In our study, 79.3% of immune-tolerant phase patients belonged to high viral load, age over 30 years, or had a history of cirrhosis or liver cancer in the family, all of whom are also eligible for expanded antiviral therapy and thus should not discontinue therapy postpartum.

While the efficacy of TDF antiviral therapy in pregnant and postpartum women with chronic HBV infection has been well-established, concerns remain regarding its long-term safety on bone and kidney health, as well as the risk of virological breakthroughs, necessitating further clinical data. Our study results demonstrate good bone and kidney safety of TDF treatment up to 144 weeks postpartum, consistent with findings by Pietro et al. (26, 27). No cases of virological breakthrough were observed during the 48-week postpartum follow-up; however, there were instances of suboptimal response and low viremia patients, adjusting the treatment regimen to extend antiviral therapy duration resulted in virological response, highlighting the potential benefits of prolonged antiviral therapy in immune-tolerant phase patients. The survival analysis in our study dynamically illustrated the kinetics of CVR achievement in pregnant women with different clinical phases of HBV infection. We found that women in the CHB group had a significantly higher hazard of achieving CVR and a shorter median time to CVR compared to those in the ITP group. This result strongly suggests that for pregnant women in the immune-tolerant phase (ITP), who typically present with exceptionally high baseline viral loads, the virological response may be comparatively slower. Therefore, clinicians should reinforce patient education and adherence monitoring for ITP women, emphasizing the necessity and importance of persisting with the full course of TDF therapy and managing expectations regarding the rate of virological response. Our data provide a rationale for adopting a more patient and persistent management strategy for this specific subgroup of pregnant women.

However, this study was limited to pregnant women who received TDF treatment in the infectious disease outpatient department of our hospital, resulting in certain sample selection limitations and a restricted sample size. Incomplete clinical data were also noted. Additionally, due to factors such as regional disparities, the generalizability of our findings to national and international populations may be subject to bias. Further prospective, multicenter, large-sample studies are required to validate these results and evaluate long-term outcomes, thereby providing more robust data to guide antiviral therapy recommendations for pregnant women in the immune-tolerant phase.

In conclusion, postpartum discontinuation of TDF therapy poses a high risk of rebound in immunotolerant pregnant women. Continuing TDF therapy postpartum up to 144 weeks demonstrates both efficacy and safety. Immunotolerant pregnant women should therefore receive long-term antiviral management consistent with HBV-infected populations to reduce mother-to-child transmission rates. Postpartum continuation of antiviral therapy without cessation provides sustained virological suppression, reduces the risk of disease progression, and ultimately benefits patients.

## Conclusion

Postpartum discontinuation of TDF poses significant risks for immunotolerant pregnant women, whereas continuing TDF treatment for 144 weeks postpartum demonstrates favorable antiviral efficacy, bone and renal safety profiles.

## Data Availability

The original contributions presented in this study are included in this article/supplementary material, further inquiries can be directed to the corresponding author.
